# Being yourself is a defect: analysis of documented rights violations related to sexual orientation, gender identity and HIV in 2022 using the REAct system in six eastern European, Caucasus and Central Asian countries

**DOI:** 10.1002/jia2.26311

**Published:** 2024-07-19

**Authors:** Oksana Kovtun, Elvira Tilek kyzy, Nadira Masiumova

**Affiliations:** ^1^ Alliance for Public Health Kyiv Ukraine; ^2^ Eurasian Coalition on Health Rights, Gender and Sexual Diversity Tallinn Estonia

**Keywords:** human rights, men who have sex with men, transgender people, eastern Europe, Caucasus and central Asia, sexual orientation and gender identity

## Abstract

**Introduction:**

Removing legal barriers to HIV services is crucial for the global 2030 goal of ending the HIV and AIDS epidemic, particularly in eastern Europe, the Caucasus and central Asia. Despite state commitments to uphold human rights, gay, bisexual and other men who have sex with men (gbMSM), along with transgender people (TP) still face stigma and discrimination. This article presents an analysis of rights violations based on sexual orientation and gender identity (SOGI) and HIV reported in 2022 across six countries, highlighting features and their links to legislation and law enforcement practices.

**Methods:**

We examined documented cases of rights violations among gbMSM and TP in Armenia, Kazakhstan, Kyrgyzstan, Tajikistan, Uzbekistan and Ukraine in 2022 using the REAct system, a tool for documenting and responding to rights violations against key populations. Initially, we employed directed content analysis based on Yogyakarta Principles to analyse narratives of violations. A codebook was developed through contextual, manifest and latent coding, with themes, categories and codes converted into quantitative variables for statistical analysis. Descriptive statistics were used to identify the characteristics of violations.

**Results:**

A total of 456 cases of rights violations related to SOGI and HIV were documented, ranging from 22 cases in Tajikistan to 217 in Ukraine. Most violations concerned gbMSM (76.5%), with one‐fifth involving TP, predominantly transgender women. Complex violations with multiple perpetrators or infringements were documented in Armenia and central Asia. Privacy rights were commonly violated, often through outing. Cases of violations of the right to the highest attainable standard of health (13.6%) and protection from medical abuses (2.6%) were also documented. Other rights violations were sporadic, with each country exhibiting distinct patterns of violated rights and types of violations. In Ukraine, the full‐scale war in 2022 influenced the nature of documented cases, reflecting the challenges faced by gbMSM and TP.

**Conclusions:**

Monitoring rights violations proved effective for assessing the situation of gbMSM and TP, particularly in the insufficiently studied and diverse eastern Europe, Caucasus and central Asia regions. As rights violations are linked to both legislation and law enforcement practices, comprehensive interventions to minimize structural and interpersonal stigma are essential.

## 1 INTRODUCTION

Eliminating legal barriers to HIV services is crucial for achieving the global goal of ending the HIV and AIDS epidemic by 2030 [1, 2], particularly among gay, bisexual and other men who have sex with men (gbMSM) and transgender people (TP, including transgender men and transgender women) in eastern Europe, Caucasus and central Asia (EECCA). UNAIDS data indicate a rapid 49% increase in new HIV cases in the region from 2010 to 2022 [3], with gbMSM and TP accounting for 22.0% and 0.8%, respectively, among the 160,000 people who newly acquired HIV [4]. The median HIV prevalence was 4.3% among gbMSM and 1.7% among TP [3]; however, data for the EECCA region are limited [5, 6, 7].

Despite states’ commitments to uphold the rights of gbMSM and TP [8] and contribute to the Sustainable Development Goals [9], community representatives continue to face stigma and discrimination. Studies partially indicate a direct link between rights violations and HIV seropositivity among gbMSM and TP [10, 11]. Structural stigma, marked by the absence of protective policies, and interpersonal and individuated stigma, along with direct experience of right violations, lead to risky behaviours like unprotected sex and drug use [12, 13, 14]. Stigma complicates access to medical, social and legal services [15, 16], reducing readiness for HIV prevention [17] and testing [11, 18]. Intersectional identities (intersectionality [19]) exacerbate stigmatization and discrimination, particularly for gbMSM and TP involved in sex work or living with HIV [20, 21], highlighting the complexity of challenges faced by these populations. In countries of the former Soviet Union, gbMSM and TP rights protection remains inadequate [22], leading to ongoing rights violations [23]. International indices highlight a high prevalence of homophobia and transphobia [24, 25], although variations exist within the region [23, 24, 25, 26, 27]. For example, Uzbekistan and Turkmenistan still criminalize consensual same‐sex relations between men.

Efforts to eliminate stigma and discrimination based on sexual orientation and gender identity (SOGI), alongside gender‐equitable HIV programmes, effectively restrain HIV spread [8, 28]. Rights monitoring by non‐governmental organizations (NGOs) and community‐based organizations enhances protection [29] and is vital for developing democratic, evidence‐based policies [30] and improving HIV programme design and effectiveness [31].

In this article, we present the results of the analysis of documented rights violations among gbMSM and TP across six EECCA countries in 2022, highlighting the specific patterns and the impact of laws and enforcement. As rights violations among key populations (KPs) in the region are understudied, our findings contribute valuable evidence crucial for eliminating legal barriers and advocating for the health rights of the gbMSM and TP.

## 2 METHODS

### 2.1 REAct overview

Developed by Frontline AIDS, the “Rights—Evidence—Action” (REAct) is a community‐based tool to monitor and respond to human rights violations [32]. Implemented in Ukraine by the International Charitable Foundation “Alliance for Public Health” (APH) since 2019, and funded by the Global Fund to Fight AIDS, Tuberculosis and Malaria, it was adopted by 63 NGOs in 18 Ukrainian regions by 2022. In 2022, the Eurasian Coalition on Health, Rights, Gender and Sexual Diversity (ECOM) expanded REAct to Armenia, Kazakhstan, Kyrgyzstan, Tajikistan and Uzbekistan, focusing on gbMSM and TP rights violations [33].

REAct enables KPs to report violations to a case documenter (“REActor”) in person or online [34], promoted by community activists through NGOs and social media. Regional teams, including KPs and NGOs staff, register cases upon receiving complaints. REAct criteria require cases to be individual‐specific (in Ukraine), involve actual rights violation, be reported by survivors or close associates, relate to the survivor's HIV status or KP and be distinct from previously documented incidents. Non‐violations include routine help requests or unrelated scenarios to HIV status or SOGI. REActors document each case through an interview, detailing the violation's location, timing, perpetrators and witnesses, reasons, and nature, aligning with recommended guidelines [35, 36]. Following the interview, REActors develop action plans, offer consultations, psychological support and refer the survivor to legal or specialized services, while monitoring each case and logging them into an information system.

All REAct operations follow standardized guidelines and tools from Frontline AIDS [32], ensuring regional consistency and tool adaptation to local languages and data collection needs, enhancing documentation effectiveness and reliability.

### 2.2 Population and setting

In February 2023, we conducted a secondary data analysis of gbMSM and TP (transgender men and transgender women) rights violation cases documented in the REAct system across six countries in 2022: Armenia (Caucasus), Kazakhstan, Kyrgyzstan, Tajikistan, Uzbekistan (central Asia) and Ukraine (eastern Europe). Data on the legal and social dimensions of gbMSM and TP rights and the HIV landscape in these countries are annually compiled by ECOM [23, 37–39].

Our analysis used a dataset provided by REAct coordinators in Ukraine (APH) and the remaining five countries (ECOM), ensuring no personalized information about survivors, only descriptions of gbMSM and TP right violation cases. Coordinators reviewed case descriptions to guarantee adherence to ethical standards and criteria before handing them to the research team. All cases were checked for discrimination based on SOGI or HIV, and no duplicates were found.

The analysis included cases of rights violations against survivors self‐identifying as gbMSM, TP and lesbian, bisexual and queer women, as well as community‐wide violations (without specifying individual SOGI) like hate speech from media, political or religious figures. Additionally, cases involving people living with HIV who identify as gbMSM or TP and face rights violations related to their HIV status were included.

### 2.3 Data processing and analysis

To convert qualitative data into quantitative, a direct content analysis approach [40] was used, with the Yogyakarta Principles on the application of international human rights law in relation to SOGI [41] as a foundational framework to categorize human rights and potential violations related to gbMSM and TP issues. The themes, categories and codes identified formed the basis for a preliminary codebook.

Each case description was analysed to identify key elements indicating specific human rights violations and characteristics of the violation, such as the type of perpetrator. Contextual coding [42] helped understand the cultural context of each case [43]. Manifest coding was used to identify explicit mentions of violations, while latent coding helped detect subtler indicators [44, 45].

During the coding stage, the codebook was updated for accuracy and completeness. Initially, one researcher (OK) created a preliminary codebook. For reliability, three researchers (OK, ET and NM) independently coded 10% of cases. After primary coding, discrepancies were resolved through consensus discussion. The remaining cases were coded by two researchers (OK and ET), with ongoing codebook refinement. The final codebook, reviewed and approved by NM, is presented in File S1.

Themes, categories and codes were converted into quantitative variables for statistical analysis. For example, if torture was identified, the corresponding variable was assigned as “1”; otherwise, it was assigned a “0.” Descriptive statistics included variables such as survivors’ SOGI, involvement of minors, violation basis, number and type of perpetrators, and violation type. One case could involve multiple perpetrators, violation types and violated rights. Instances of name‐calling and verbal abuse related to SOGI or HIV were not classified separately, as they were common in documented cases and were included in the variable “Hate speech and public incitement by individuals” if the case was limited to that aspect.

Data processing and analysis were performed using Microsoft Excel and IBM SPSS Statistics 28.0 (IBM SPSS Statistics for Windows, Version 28.0. Armonk, NY: IBM Corp).

### 2.4 Ethical considerations

During violation reporting and response, principles of privacy, confidentiality and voluntary participation were maintained. REActors were trained in methodologies, human rights, legislation and providing appropriate support to minimize re‐traumatization risks. Private communication with survivors included thorough threat and risk assessment to address potential issues such as family retaliation or punitive laws against homosexual relationships, with mitigation strategies aligned with international protocols [46]. Paper documentation was destroyed post‐entry for confidentiality, and a security plan [47] was implemented for data protection.

Survivors reporting rights violations through REAct were asked to provide three separate written informed consents tailored to each purpose: (1) to document the case and participate in the interview; (2) to use the de‐personalized case description for advocacy and research; and (3) to use personal data for legal submission if they chose to involve a lawyer. If a survivor declined certain consents, they still received appropriate services according to their agreed participation level. For example, if a survivor declined advocacy and research consent but agreed to data sharing with a lawyer, they received legal assistance without their case being used for advocacy or analysis. If a survivor consented only to project participation, their case was used solely for programme monitoring by the REAct coordinator, including counting documented cases, assessing assistance effectiveness, and determining resolution and resource use. Informed consent forms [48] were developed by Frontline AIDS [32] and adapted for each country.

In this study, we analysed textual descriptions of rights violations collected by the REAct system, initially designed for reporting and responding to such violations. The analysis, limited to narrative descriptions without identifiable information, was determined not to involve human subjects research, and a full ethical review was waived (Institutional Review Board No. 1 of the charity organization “Ukrainian Institute on Public Health Policy,” IRB#00007612, FWA #00029648). All cases included were verified by REAct coordinators from APH and ECOM to ensure survivor consent for research use and the absence of confidential information. No refusals were recorded; thus, all documented cases reported for 2022 from each country were included in the analysis.

## 3 RESULTS

In 2022, a total of 456 gbMSM and TP rights violation cases were documented across six countries: 217 in Ukraine, 80 in Armenia, 79 in Uzbekistan, 32 in Kyrgyzstan, 26 in Kazakhstan and 22 in Tajikistan (Table 1). Most survivors were gbMSM (76.5%), while TP constituted 20%, primarily transgender women. Nearly, all cases (98.5%) involved SOGI‐based violations, sometimes intersecting with HIV status. Kyrgyzstan (43.8%) and Kazakhstan (26.9%) reported higher proportions of HIV‐related cases.

**Table 1:** Rights violation cases by countries, 2022

		Central Asia					
	AM	KZ	KG	TJ	UZ	UA	Overall
Characteristic	%	%	%	%	%	%	%
**Total cases (*N*)**	**80**	**26**	**32**	**22**	**79**	**217**	**456**
**SOGI of survivor**							
MSM, gay and bisexual men	52.5	61.5	93.8	63.6	87.3	82.0	76.5
Transgender men	3.7	−	6.2	−	−	6.0	4.0
Transgender women	37.5	23.1	−	36.4	3.8	12.0	16.0
Lesbian, bisexual and queer women	3.8	−	−	−	1.3	−	0.9
Entire LGBT community (without specific SOGI)	2.5	15.4	−	−	7.6	−	2.6
**Cases involving minors**	**6.3**	−	**3.1**	−	**1.3**	0.5	1.8
**Basis of violation**							
SOGI	100.0	84.6	93.8	100.0	100.0	99.5	98.5
HIV	3.8	26.9	43.8	9.1	6.3	3.2	8.3
SW	8 10.0	11.5	6.3	13.6	6.3	−	4.6
**Combined basis of violation**							
SOGI only	86.3	61.5	50.0	77.3	87.3	96.8	87.1
SOGI and HIV	3.8	11.5	37.5	9.1	6.3	2.8	6.8
SOGI and SW	10.0	11.5	6.3	13.6	6.3	−	4.6
HIV only	−	15.4	6.3	−	−	0.5	1.5
**Cases with multiple violated rights**	**45.0**	**42.3**	**43.8**	**77.3**	**72.2**	33.6	45.6
**Cases with multiple types of violations**	**60.0**	**53.8**	**78.1**	**86.4**	**84.8**	47.0	60.3
**Cases involving group perpetrators**	**47.5**	**50.0**	**40.6**	**68.2**	**60.8**	40.1	46.9
**Cases with multiple types of perpetrators**	**26.3**	**19.2**	**15.6**	**22.7**	**19.0**	8.3	15.1
**Type of perpetrators**							
Unidentified person	30.0	26.9	15.6	13.6	34.2	23.5	25.7
Police	18.8	15.4	25.0	63.6	31.6	11.5	20.0
General healthcare facility	17.5	−	28.1	4.5	2.5	8.8	9.9
Relative	13.8	7.7	6.3	4.5	15.2	6.5	9.2
Military	−	−	−	13.6	−	11.5	7.7
Employer	2.5	11.5	12.5	−	3.8	7.4	6.1
Colleague, classmate	3.8	−	6.3	−	2.5	7.4	5.0
Service industry worker	2.5	−	−	−	−	9.2	4.8
Neighbour	1.3	−	−	−	7.6	5.5	4.2
Sexual partner	2.5	−	−	9.1	8.9	3.7	4.2
HIV/AIDS healthcare facility	2.5	15.4	6.3	4.5	2.5	1.8	3.3
Government official	3.8	7.7	3.1	4.5	−	3.2	3.1
SW’ client	8.8	11.5	6.3	−	1.3	−	2.9
Landlord	−	7.7	−	4.5	−	4.1	2.6
Media, blogger	6.3	7.7	3.1	−	3.8	−	2.4
Educational institution	1.3	3.8	3.1	−	1.3	0.9	1.3
Acquaintance	1.3	−	−	−	1.3	1.8	1.3
Religious leader	1.3	3.8	3.1	−	2.5	−	1.1
State lawyer	−	−	−	−	3.8	0.5	0.9
Non‐governmental organization	−	3.8	−	−	1.3	0.5	0.7
**Cases involving deceptive dating**	**2.5**	**23.1**	**18.8**	**9.1**	**40.5**	**5.1**	**12.9**

Abbreviations: AIDS, acquired immunodeficiency syndrome; AM, Armenia; HIV, human immunodeficiency virus; KG, Kyrgyzstan; KZ, Kazakhstan; LGBT, lesbian, gay, bisexual and transgender people; MSM, men who have sex with men; SOGI, sexual orientation and gender identity; SW, sex work; TJ, Tajikistan; UA, Ukraine; UZ, Uzbekistan.

Almost half of the cases included multiple rights violations (33.6% in Ukraine to 77.3% in Tajikistan), with 60.3% involved multiple types of violations. Group perpetrators were involved in 46.9% of cases, notably in Uzbekistan (60.8%) and Tajikistan (68.2%). Deceptive dating (where a non‐gbMSM or TP person connects via social media for a date to exploit and manipulate information about counterpart's SOGI) mostly seen in Uzbekistan (40.5%) compared to 12.9% overall.

Perpetrators were commonly unidentified (25.7%) or law enforcement officers (20.0%), with the latter being particularly prominent in Tajikistan (63.6%). In Armenia, violations by clients of sex workers (SWs) were substantial (8.8%), while Kazakhstan and Kyrgyzstan reported more violations by healthcare facilities. Military involvement in violations was noted in Tajikistan (13.6%) and Ukraine (11.5%). Ukraine and Tajikistan had no cases involving clients of SWs, religious leaders or media representatives as perpetrators, unlike other countries. In Ukraine, colleagues/classmates and acquaintances were significant perpetrators, as were service industry workers (9.2%) compared to 4.8% across other countries.

The analysis of gbMSM and TP rights violations in REAct across six countries revealed disparities in the nature and frequency of these violations (Table 2). Among these, 38.2% involved infringements of the right to privacy, primarily through outing, while 35.3% were linked to the right to personal security. Physical violence, the most prevalent form of personal security violation, ranged from 11.5% in Kazakhstan to 30.4% in Uzbekistan. Uzbekistan notably had a third of the cases involving property damage and material harm related to the right to recognition before the law. Ukraine had cases related to denial of services due to transgender transition, accounting for 2.3% of all cases. Torture by law enforcement was documented in 36.4% of cases in Tajikistan, higher than the overall average of 4.8%.

**Table 2:** Types of rights violations based on SOGI and HIV by countries, 2022

		Central Asia					
	AM	KZ	KG	TJ	UZ	UA	Overall
Right/violation type	%	%	%	%	%	%	%
**Privacy**	**31.3**	**38.5**	**43.8**	**54.5**	**74.7**	**24.9**	**38.2**
Disclosure or threat of disclosing SOGI (outing)	22.5	19.2	28.1	23.7	54.4	19.4	27.0
Extortion of money	5.0	19.2	18.8	31.8	31.6	6.5	13.4
Unauthorized access to private correspondence	2.5	7.7	9.4	18.2	15.2	6.9	8.3
Unauthorized home or personal inspection	1.3	−	−	4.5	2.5	3.7	2.6
Coercion to disclose partner information	2.5	3.8	9.4	13.6	3.8	−	2.6
Disclosure or threat of disclosing HIV status	1.3	3.8	6.3	−	2.5	0.9	1.8
Criminalization of MSM		−	−	−	7.6	−	1.3
Criminalization of HIV transmission	1.3	−	−	−	2.5	−	0.7
**Security of the person**	**47.5**	**19.2**	**25.0**	**27.3**	**46.8**	**30.9**	**35.3**
Physical violence	31.3	11.5	15.6	13.6	30.4	23.0	24.1
Domestic violence	11.3	7.7	3.1	9.1	15.2	6.5	8.8
Sexual violence and harassment	7.5	−	9.4	4.5	2.5	0.9	3.1
Coercion to use drugs or alcohol	1.3	−	−	−	−	0.5	0.4
**Recognition before the law**	**15.0**	**11.5**	**6.3**	**22.7**	**31.6**	**13.8**	**16.9**
Property damage and material harm	7.5	11.5	3.1	22.7	31.6	9.2	13.2
Denial to process documents related to transgender transition	5.0	−	3.1	−	−	2.8	2.4
Denial of social services based on transgender transition	−	−	−	−	−	2.3	1.1
Coercion to conceal SOGI	2.5	−	−	−	2.5	−	0.9
**Freedom from torture and cruel, inhuman or degrading treatment or punishment**	**3.8**	**3.8**	**12.5**	**50.0**	**22.8**	**12.4**	**14.0**
Other abuses of authority by security and law enforcement	3.8	3.8	9.4	13.6	17.7	12.0	11.0
Force, torture or cruelty by security and law enforcement	−	−	6.3	36.4	6.3	3.2	4.8
**Highest attainable standard of health**	**20.0**	**15.4**	**34.4**	**9.1**	**5.1**	**11.5**	**13.6**
Demeaning conduct in HCF	13.8	3.8	31.3	4.5	2.5	6.9	8.8
Denial to provide medical services	13.8	−	18.8	−	−	4.6	5.9
Disclosure of SOGI medical data	2.5	3.8	−	4.5	1.3	0.9	1.5
Disclosure of HIV medical data	−	11.5	−	4.5	1.3	−	1.1
Denial of medical services related to transgender transition	1.3	−	−	−	−	1.8	1.1
Denial to prescribe PrEP	−	−	−	−	−	1.8	0.9
Extortion of payment for medical services, whether free or paid	−	−	6.3	−	1.3	0.5	0.9
Denial of hepatitis or STI treatment	−	−	3.1	−	−	0.5	0.4
Denial to prescribe ART	1.3	−	−	−	−	−	0.2
**Effective remedies and redress**	**12.5**	**15.4**	**3.1**	**9.1**	**8.9**	**5.1**	**7.7**
Denial to provide legal assistance	12.5	15.4	3.1	9.1	8.9	5.1	7.7
**Work**	**5.0**	**11.5**	**12.5**	−	**3.8**	**8.8**	**7.2**
Employment termination or denial	2.5	11.5	12.5	−	3.8	5.1	5.0
Workplace bullying	3.8	−	3.1	−	1.3	5.5	3.7
Denial to pay wages	−	−	3.1	−	−	0.9	0.7
HIV certificate demand during employment	−	3.8	3.1	−	−	−	0.4
**Adequate housing**	**2.5**	−	**3.1**	**4.5**	**5.1**	**10.1**	**6.6**
Eviction or forced eviction	2.5	−	3.1	4.5	5.1	7.8	5.5
Denial of hotel and shelter services	−	−	−	−	−	2.3	1.1
**Freedom from arbitrary deprivation of liberty**	**2.5**	**3.8**	**6.3**	**27.3**	**13.9**	**2.3**	**5.9**
Illegal arrest or detention	2.5	−	6.3	27.3	6.3	2.3	4.4
Coercion to provide self‐incriminating statements	−	3.8	−	9.1	7.6	−	2.0
**Freedom of opinion and expression**	**6.2**	**15.4**	**6.3**	−	**7.6**	**1.8**	**4.6**
Hate speech in media and public figures	6.3	15.4	3.1	−	6.3	0.5	3.5
Hate speech and public incitement by individuals	−	−	3.1	−	1.3	1.4	1.1
**Equality and non‐discrimination**	**2.5**	−	−	−	−	**6.0**	**3.3**
Service denial or degrading conduct in the private sector	2.5	−	−	−	−	6.0	3.3
**Freedom of movement**	**2.5**	−	−	−	**8.9**	**2.8**	**0.3**
Forced departure from city/country	2.5	−	−	−	8.9	−	2.0
Denial to cross borders	−	−	−	−	−	2.8	1.3
**Protection from medical abuses**	−	**11.5**	**3.1**	**9.1**	**5.1**	**0.9**	**2.6**
Forced anal examinations	−	−	−	9.1	5.1	−	1.3
Forced HIV testing	−	7.7	3.1	−	2.5	−	1.1
Forced medical treatment	−	3.8	−	−	−	0.9	0.7
**Freedom of peaceful assembly and association**	**1.3**	**11.5**	−	**4.5**	**2.5**	**1.8**	**2.4**
Obstacles in the work of non‐governmental organizations	−	11.5	−	4.5	2.5	1.4	2.0
Obstacles in conducting meetings	1.3	−	−	−	−	0.5	0.4
**Education**	**1.3**	−	**3.1**	−	−	**3.2**	**2.0**
Bullying in educational institutions	1.3	−	3.1	−	−	3.2	2.0
**Life**	**5.0**	**7.7**	−	−	**1.3**	**0.5**	**1.8**
Murder or attempted murder	5.0	7.7	−	−	1.3	0.5	1.8
**Found a family**	−	−	**3.1**	−	**3.8**	−	**0.9**
Coercion into marriage	−	−	3.1	−	3.8	−	0.9
**Participate in public life**	−	−	**3.1**	−	−	**0.9**	**0.7**
Denial of employment or dismissal from public service	−	−	3.1	−	−	0.5	0.4
Denial of employment in security and law enforcement	−	−	−	−	−	0.5	0.2
**Social security and to other social protection measures**	−	−	−	−	−	**0.9**	**0.4**
Denial of benefits or state assistance	−	−	−	−	−	0.9	0.4

Abbreviations: AM, Armenia; ART, antiretroviral therapy; HCF, healthcare facility; KG, Kyrgyzstan; KZ, Kazakhstan; MSM, men who have sex with men; PrEP, pre‐exposure prophylaxis; SOGI, sexual orientation and gender identity; STI, sexually transmitted infection; TJ, Tajikistan; UA, Ukraine; UZ, Uzbekistan.

Violations concerning the denial of the highest attainable standard of health were most evident in Kyrgyzstan (34.4%) and Armenia (20.0%), primarily involving demeaning conduct in healthcare facilities. Ukraine documented cases of denial in prescribing pre‐exposure prophylaxis (PrEP), a notable divergence from other countries. In Kazakhstan, disclosure of HIV medical data occurred in one out of every 10 cases, a proportion substantially higher than the 1.1% average across all countries. Cases of forced anal examinations were recorded in Tajikistan and Uzbekistan, and forced HIV testing was noted in Kazakhstan, Kyrgyzstan and Uzbekistan. Forced medical treatment was documented in Kazakhstan and Ukraine.

Other human rights violations of gbMSM and TP were sporadically documented. Tajikistan had a high incidence of illegal arrest (27.3%), while Kazakhstan showed a notable proportion of denial of legal assistance and restrictions on freedom of expression (15.4%), as well as obstacles in NGO functioning (11.5%). Ukraine reported cases including denial of hotel and shelter services, forced eviction, a proportion of which were double those in other countries, employment denial in security and law enforcement, and denial of benefits or other state assistance. Ukraine's right to freedom of movement violations was primarily due to the denial of border crossing, while Uzbekistan and Armenia documented forced departures. Murders or attempted murders were recorded in several countries, totalling eight cases, while coerced marriage cases were documented in Uzbekistan and Kyrgyzstan.

## 4 DISCUSSION

In this study, we analysed human rights violations related to SOGI and HIV recorded by REAct system in six EECCA countries. These findings highlight challenges faced by gbMSM and TP and provide insights for HIV programming and efforts to combat stigma, discrimination and legal barriers. The results deepen understanding of structural and interpersonal stigma, emphasizing violations of rights based on SOGI rather than solely on HIV status [49], and revealing informal behaviour practices often overlooked in policy analyses [13].

Distinct patterns of violations across countries underscore the necessity for culturally and regionally tailored approaches. In the Caucasus and central Asia, similar to other countries with conservative social and religious norms [50], restrictions on same‐sex relationships contribute to SOGI‐based stigma and discrimination [5, 51], stemming from the Soviet era [52] where homosexuality was criminalized and pathologized [53]. Cases of unauthorized access to personal communication, outing, physical violence, forced departure primarily by family members and the use of hate speech illustrate this issue. Uzbekistan, where same‐sex relationships have been criminalized since 1994, faces challenges aligning with studies in other countries with similar conditions [11]. Decriminalization is imperative to potentially slow the HIV epidemic and promote increased usage of healthcare services, as evidenced by comparative research on regions with and without criminalization [54]. Armenia decriminalized homosexuality and same‐sex relationships in 2003, later than most regional countries, but the political environment remains unfriendly, reinforcing prejudices and exacerbating manifestations of homophobia and transphobia [55]. Transgender women in Armenia remain the most marginalized and vulnerable to violence, consistent with previously published data [56]. Nonetheless, gbMSM and TP movement has emerged in the country in recent years [57], providing avenues for expression despite lacking support from the government, politicians and media [56].

Deceptive dating, where gbMSM and TP are exploited and manipulated through deceitful online interactions, is a frequent form of exploitation also noted in other studies [58]. Perpetrators lure survivors into social network, leading to meetings that escalate into violence, illegal arrests or extortion to conceal SOGI. Perpetrators often falsely claim to be law enforcement officers to deter reporting to the police. Our findings, consistent with other studies [59], show that gbMSM and TP are affected by “revenge porn,” where explicit content is distributed without consent, leading to outing and discrimination, issues likely more prevalent than among heterosexuals [60]. In environments of high SOGI‐based stigma and police indifference, online platforms become vital for community interactions and partner‐seeking [11, 17], underscoring the need for awareness of safe online practices [61] and the risks of sharing personal data. Deceptive dating often targets gbMSM and TP engaged in sex work, highlighting the need for evidence‐based interventions to address intersectional stigma [21].

Violations of health rights and protection from medical abuses were common, including forced anal examinations, outing and coercion to disclose of HIV status in countries with inadequate HIV legislation enforcement [54]. Epidemiological procedures in Kazakhstan [62] and Kyrgyzstan [26] led to human rights violations, emphasizing the need for enhanced healthcare provider training [50]. In Uzbekistan, health authorities must report HIV transmission to law enforcement [26], leading to partner disclosure and administrative consequences [63]. Criminalizing unintentional HIV transmission in Armenia [64] and its presence in Uzbekistan have led to rights violations, as our findings show. Documented cases of rights violations by healthcare staff ranged from demeaning treatment to service refusal, particularly in primary healthcare settings, necessitating tolerance training and inclusive medical education.

Russia's full‐scale war against Ukraine since 24 February 2022 reshaped reported rights violations in Ukraine, with reduced activity of ultra‐conservative movements, rejection of locally prepared homophobic appeals, ratification of the Istanbul Convention [65], and improved attitudes towards the gbMSM and TP among Ukrainians [66]. Wartime context facilitated citizens’ petitions and the inclusion of a law on civil partnership in Ukraine's Recovery Plan [23]. However, in 2022, Ukraine documented a higher proportion of housing‐related violations based on SOGI, including instances of denial of temporary accommodation in hotels and shelters, within the context of dynamic internal migration resulting from the war [67]. Military‐related violations increased, driven by document checks under martial law [68]. Despite this, violations commonly seen in other countries, such as unlawful arrests or physical force during detention, were less frequently documented. Border crossing challenges affected transgender women without proper certificates [69] or border guards’ unfamiliarity with TP crossing procedures [70], emphasizing the need for further efforts to ensure TP rights during wartime.

In Ukraine, TP faced refusals of social services and document issuance related to their transition. Unlike the five other countries [71], Ukraine has implemented a legal procedure for transgender transition, avoiding compulsory surgeries with only an age criterion of 14 [72]. However, authorized gender marker changes based on current residence during martial law were inconsistently known by state agency personnel, resulting in violations. Anti‐discrimination legislation and established transition producers in Ukraine increase service‐seeking by TP [16], raising the likelihood of rights violations. Documented cases of denied PrEP prescriptions for gbMSM and TP could be linked to its popularity and availability since 2018 [73], contrasting with countries beginning PrEP implementation in 2021 [74]. Despite training programmes for medical [75] and law enforcement personnel, further development within HIV programmes is needed to minimize rights violations.

### 4.1 Limitations

We acknowledge limitations in our analysis, including potential underreporting of rights violations among gbMSM and TP due to survivor's lack of awareness about REAct or choice not to report, and reliance on reports from those willing to seek help [16, 76]. Research shows high tolerance towards domestic violence among TP [77], potentially due to a lack of education and recognition of rights violation. High levels of internalized stigma reduce engagement, visibility [78, 79], awareness of services and willingness to participate in HIV prevention [80, 81, 82], thereby diminishing support for protecting their rights and participating in actions against discrimination and violence [83]. In the Caucasus and central Asia, MSM show lower self‐acceptance compared to the European part of the EECCA region [83], with limited data for TP [81]. Data show higher levels of stigma and discrimination than documented, with 36% of gbMSM in Ukraine [84] and 48% of MSM and 1% of transgender women in Yerevan, Armenia, reported feeling ashamed about their SOGI [85].

The distribution of registered cases across countries varies due to different REAct implementation periods and country‐specific factors. Ukraine introduced the system in 2019, while other countries under ECOM did so in 2022, leading to varying effectiveness levels based on monitoring team experience and capability. Ukraine's sustained initiatives highlight the system's values, as REAct recorded 108 violations against gbMSM in 12 regions in its first year [86], increasing to 217 by 2022. The number of documented cases can be partly explained by its more supportive legal environmental and reduced structural barriers [79], as Ukraine has the highest level of legal protection for these communities [23]. Reduced structural stigma encourages more active participation of gbMSM and TP in research [5, 10], engagement with NGOs [14], contrasting with Tajikistan's legislative restrictions on community‐based NGOs registration [23], potentially leading to fewer reported violations and increased HIV transmission risk as found in a recent African study [54]. Comparing estimated numbers of gbMSM and TP reveals disparities across countries, making direct comparisons of cases based on absolute numbers unreliable. Ukraine reported the highest number of cases, with 152,267 gbMSM [87], while other countries varied from 3000 to 62,000 [37]. In Ukraine, the estimated number of TP stands at 9963 people [87], while in Armenia and Kyrgyzstan, it does not exceed 1000 TP [38].

Differences in implementing REAct system between the Caucasus and central Asia region and Ukraine have contributed to diverse survivor profiles. ECOM focused on gbMSM and TP rights, while other organizations documented violations against other KPs [34]. The Ukrainian project included all KPs, addressing gbMSM and TP among others. A recent study found that 28.6% of male and transgender SWs faced violence, with only 7.9% seeking help [88], and violations against gbMSM and TP in sex work might have been documented as SWs’ violations, contributing to a lower sexual abuse rate. Unlike the CCA countries, Ukraine lacked documented violations against the entire LGBT community, possibly due to criteria for documenting violations. Data likely underrepresent lesbian, bisexual and queer women, as they are not recognized as KP [27, 89–92], but analysed cases show their rights violations and vulnerability to HIV acquisition [93].

While Ukraine records the highest number of violations, the REAct's coverage may be incomplete due to expanding rights documentation projects [65, 94, 95]. Independent programmes plan a coordinated effort in 2024 to overcome legal barriers affecting KPs [96, 97], promising more comprehensive data.

## 5 CONCLUSIONS

Our study in six EECCA countries provides crucial insights into the rights violations among gbMSM and TP in 2022. Monitoring rights violations based on SOGI and HIV is effective in identifying legal issues, stigma and discrimination, informing recommendations to enhance HIV responses. Our findings underscore the imperative to address stigma and discrimination not only at individual level but also within community and structural contexts, emphasizing the need for comprehensive interventions.

## COMPETING INTERESTS

The authors have declared that no competing interests exist.

## AUTHORS’ CONTRIBUTIONS

OK conceptualized and drafted the manuscript, led the content analysis, conducted statistical analysis and interpreted the data. ETk and NM contributed data for the analysis, actively participated in discussions, and critically reviewed and edited the manuscript. The authors assume responsibility for the integrity and accuracy of the data analysis. The final version of the manuscript has been read and approved by all authors.

## Supporting information


**File S1**: Codebook. The final version of the coding book used in the analysis presented in the article.

## Data Availability

The data that support the findings of this study are available on request from the corresponding author. The data are not publicly available due to privacy or ethical restrictions.
