# Supplementary material for: Being yourself is a defect: analysis of documented rights violations related to sexual orientation, gender identity and HIV in 2022 using the REAct system in six eastern European, Caucasus and Central Asian countries
Source: J Int AIDS Soc. 2024 Jul 19;27(Suppl 3):e26311. doi: 10.1002/jia2.26311 (PMC11258482; doi:10.1002/jia2.26311)
Supplement: Supplementary file 1 — File S1: Codebook. The final version of the coding book used in the analysis presented in the article. [file JIA2-27-e26311-s001.docx]

| **Theme** | **Category** | **Code** | **Description** | **Examples** |
| --- | --- | --- | --- | --- |
| **Characteristics of survivor** | SOGI | MSM, gay, and bisexual men | Refers to survivors who are men who have sex with men, gay, or bisexual | *"...a young man met another man, and over time, a relationship developed between them...", "...the applicant is a gay man..."* |
|  |  | Transgender people | Survivors who identify with a gender different from the one assigned at birth, encompassing both transgender men and women | *"...the survivor is a transgender individual...", "the applicant wants to undergo gender reassignment surgery..."* |
|  |  | Transgender women | Specifically refers to survivors assigned male at birth but who identify and live as women | *"...the transgender woman had to seek help..."* |
|  |  | LGBT community | Cases where violations target the LGBT community collectively, without specifying individual identities within the community | *"...discloses information collected without the consent of LGBT people and passes it to law enforcement..."* |
|  |  | Lesbian, bisexual and queer women | Survivors identifying as lesbian, bisexual, or queer women | *"...the survivor is a bisexual woman..."* |
|  | Age | Minors | Survivors who are legally minors | *"...frequent disputes occurred at the home of a minor who is gay...", "...a sexual contact between two guys, aged 16 and 18, was recorded... following its dissemination, they were subjected to beatings..."* |
| **Characteristics of violation** | Basis of violation | SOGI | Violations motivated primarily by the survivor's sexual orientation or gender identity | *"...acquaintances accidentally discovered he was gay and began to mock and blackmail him..."* |
|  |  | HIV | Violations that explicitly target survivors based on their HIV status, indicating a direct link between the violation and the HIV status | *"...the AIDS center found the student's HIV status to be positive and sent his personal details to the university, which then issued a mandatory order for testing..."* |
|  |  | SOGI + HIV | Violations where both the survivor's HIV status and their SOGI are integral factors in the abuse or discrimination | *"...an epidemiologist informed the survivor of his HIV status, started insulting and humiliating him, and forced him to disclose the names and details of his partners, threatening to bring the police to his home and reveal his status and orientation to his parents if he did not bring them in for testing..."* |
|  |  | SOGI + SW | Violations at the intersection of the survivor’s SOGI and their engagement in sex work | *"...the survivor is a transgender person who provides sexual services for compensation..."* |
|  | Complex violations | Group perpetrators | Incidents involving multiple perpetrators, highlighting coordinated or group-based attacks | *"...the family accidentally discovered his homosexuality, after which he was subjected to violence by his father and older brother; he was beaten, humiliated, and mocked, and was deprived of the ability to leave the house, communicate with friends, or use the phone..."* |
|  |  | Deceptive dating | Situations involving deceit or manipulation in dating scenarios, leading to abuse or exploitation | *"...we met online... He took me along with his colleagues to the police station, where I was detained for five days and mocked...", "...met online, after exchanging intimate photos he invited me to his place... when the survivor stepped outside, a man and five others emerged from a car, seized him, struck him, and threatened to disclose his homosexuality to his neighbors..."* |
| **Type of perpetrator** |  | Government official | Any violation perpetrated by officials within governmental institutions | *"...the transgender person underwent surgery and applied for legal gender recognition in their documents; though the legal review period has passed, the staff continuously delay the approval documents..."* |
|  |  | Police | Violations carried out by police officers | *"...both partners were taken to the police station, where they were insulted and mocked with questions like 'Are you a girl or a boy?', 'Have you always been a faggot or did you become one recently?'"* |
|  |  | State lawyer | Cases involving misconduct or negligence by government-appointed legal representatives | *"...a state-appointed lawyer demanded details during the investigation about what and in which positions he did things, and during the trial, the lawyer simply sat silently... the survivor was accused of spreading a venereal disease..."* |
|  |  | Military | Violations committed by members of the armed forces | *"...guards at the checkpoint forcibly took him to the basement, where they physically and emotionally abused him..."* |
|  |  | HIV/AIDS healthcare facility | Incidents of discrimination or abuse occurring within facilities specialized in HIV/AIDS services | *"...the patient was confirmed to be HIV-positive, epidemiologists relayed the information about his HIV status and that he is MSM to the local clinic, called his wife to be tested, and informed her that her husband contracted HIV from having sex with men..."* |
|  |  | General healthcare facility | Violations by medical staff in non-specialized healthcare settings | *"...a transgender woman was beaten and her friends called an ambulance. At the hospital, the staff were indifferent, denied her a wheelchair, made discriminatory remarks, addressed her as 'faggot', and spread her personal information among all hospital staff..."* |
|  |  | Educational institution | Abuses occurring within educational environments, perpetrated by educators or institutional staff | *"...a teacher publicly humiliated a student based on intolerance...", "...teachers discriminated against him, invited his parents to the school for a discussion, and disclosed the boy's homosexual orientation to them..."* |
|  |  | Relative | Violations by family members, focusing on domestic or intra-family abuse | *"...his mother deprived him of communication means – phone and internet, did not allow him to leave the house, forbade him from communicating with friends, and threatened to call his friends' parents to disclose their orientation... The applicant's mother also prepared documents to take the survivor out of the country, intending to subject him to 'conversion therapy' sessions with a psychologist..."* |
|  |  | Sexual partner | Abuses committed by a current or former intimate partner | *"...his boyfriend forces the survivor to engage in sex work, takes the money for himself, and keeps him locked up..."* |
|  |  | Neighbor | Violations by individuals living in proximity to the survivor | *"...neighbors downloaded photos from the survivor's Instagram page, created a fake page, and marked the photos with captions stating the survivor is gay and used derogatory language... This fake page was spread throughout the village, and everyone learned about the survivor's homosexuality..."* |
|  |  | Acquaintance | Abuses by people the survivor knows socially, but not closely | *"...his acquaintances found out he was gay and began to mock and blackmail him...", "one guy from the group uses drugs and knows that the survivor is LGBT... He forced him to use drugs, threatening that if the survivor refused, he would tell everyone about his SOGI..."* |
|  |  | Colleague, classmate | Workplace or educational abuses by peers or coworkers | *"...after the video spread, his classmates started treating him badly, calling him 'a woman,' 'you dress like a girl,' and they pushed and pulled at the survivor..."* |
|  |  | SW’ client | Specific to violations by clients against sex workers | *"...after intercourse, when the client was supposed to leave, he hit the woman, broke her nose, and then continued to beat her head before fleeing..."* |
|  |  | Unidentified person | Violations by perpetrators who remain anonymous or unidentifiable | *"...two strangers approached her, attacked, and struck various parts of her body, pulled out a knife, and demanded that she leave the city because she is a transgender woman and a disgrace to them..."* |
|  |  | Landlord | Abuses committed by housing providers | *"...the dormitory administration evicted them after learning that they were living together as a gay couple..."* |
|  |  | Employer | Workplace violations | *"...the director stated that he would not tolerate 'faggots' in his institution, said he wouldn't pay them a penny, and they should be glad he hasn't disclosed the survivor's orientation..."* |
|  |  | Religious Leader | Abuses perpetrated under the guise of religious authority or within religious settings | *"...an Islamic activist uses derogatory comments about the LGBT community, inciting violence..."* |
|  |  | Media, blogger | Violations propagated by media personnel or bloggers, including defamation and incitement | *"...a blogger conducted a live stream on social media, during which he made public calls for violence, threatened to burn homosexuals, and urged others to insult them..."* |
|  |  | Service industry worker | Violations committed by individuals within the service sector | *"...a transgender woman decided to buy a gym membership, and she was told to use the men's locker room..."* |
|  |  | Non-governmental organization | Abuses occurring within or involving NGOs. | *"...a representative of [NGO name] began telling in the organization's office that the survivor is gay and that he sought treatment for a venereal disease from her..."* |

*Supplied generalized examples to human rights violations are intended to illustrate identified codes. They provide condensed descriptions of case essences, avoiding detailed disclosures to uphold confidentiality and respect for the rights and dignity of the survivors*

| **Theme** | **Category** | **Code** | **Description** | **Examples** |
| --- | --- | --- | --- | --- |
| **Type of violation** | Equality and Non-Discrimination | Service denial or degrading conduct in the private sector | Refusal to provide services or engaging in conduct that degrades individuals due to their SOGI in private sector settings (e.g., businesses, private clubs) | *A transgender woman was forced to leave a cafe after the staff refused to serve her* |
|  | Recognition before the Law | Property damage and material harm | Incidents where an individual's property is damaged or they suffer financial loss due to their SOGI | *Visitors at the gym rummaged through MSM's personal belongings in his bag left in the locker room, scattering them around, and deliberately emptied the contents of his backpack onto the floor* |
|  |  | Denial to process documents related to transgender transition | Refusal by authorities to process or update legal documents to reflect a person's gender transition | *The survivor, equipped with all the required documents for a gender change in her passport, faced rejection and derogatory treatment from passport office staff and management, who insulted her by referring to her as "bro" and callously refused assistance, even resorting to mockery* |
|  |  | Denial of social services based on transgender transition | Refusal to provide government or community social services due to an individual's transgender status | *Transgender woman faced refusal from the bank to update her passport details despite her documentary gender transition, leading to a four-month ordeal of frozen funds until resolution through intervention from the bank's main office* |
|  |  | Coercion to conceal SOGI | Forcing individuals to hide their sexual orientation or gender identity to avoid legal or social penalties | *The survivor faced coercion from his family to conceal his sexual orientation and relationships, leading to violence, strict control over his life, psychological pressure, and threats, ultimately pushing him to consider suicide* |
|  | Life | Murder or attempted murder | Actions that aim to lethally harm or result in the death of individuals based on their SOGI | *A gay man was the victim of an attempted murder during an attack in a dark alley* |
|  | Security of the Person | Physical violence | Acts of force that cause bodily harm | *A lesbian was beaten up on the street by a group of strangers* |
|  |  | Domestic violence | Acts of violence occurring within the home, perpetrated by family members or partners | *The survivor endured confinement and physical assault by his parents following his disclosure of his sexuality, seeking refuge at a friend's home and legal counsel from an NGO amidst his parents' efforts to involve the police* |
|  |  | Sexual violence and harassment | Incidents of sexual assault, harassment, or other forms of sexual misconduct | *The survivor experienced sexual violence and coercion after being lured into a situation under false pretenses, leading to a traumatic assault* |
|  |  | Coercion to use drugs or alcohol | Forcing individuals to consume drugs or alcohol as a form of control or abuse | *The survivor was coerced into consuming drugs and alcohol by acquaintances who threatened to reveal their LGBTQ+ identity if they refused, resulting in a traumatic experience and fear of further exposure* |
|  | Privacy | Disclosure or threat of disclosing SOGI (outing) | Threatening to reveal someone’s sexual orientation or gender identity without their consent, or actually disclosing it | *A student was threatened with his homosexuality being disclosed to the entire university* |
|  |  | Extortion of money | Demanding money under the threat of exposing someone's SOGI and/or HIV status | *After meeting the perpetrator online, the survivor was lured to their home, where they were coerced into a compromising situation under threat of exposing their sexual orientation, resulting in physical assault and extortion of money and belongings* |
|  |  | Unauthorized access to private correspondence | Accessing someone's personal communications without permission as a means of control or harm | *Without his consent, a colleague accessed the MSM's phone, and discovered his sexual orientation, leading to accusations and subsequent workplace discrimination* |
|  |  | Unauthorized home or personal inspection | Intrusive inspections of personal spaces or belongings without a warrant or consent | *Law enforcement authorities unlawfully detained a transgender woman and her friend, confiscating their phones, subjecting them to harassment, and conducting an unauthorized search of her apartment* |
|  |  | Coercion to disclose partner information | Forcing individuals to reveal information about their romantic or sexual partners | *The survivor was interrogated by police officers who coerced them into disclosing their sexual partners under false pretenses, to incriminate the victim for allegedly infecting others with HIV* |
|  |  | Disclosure or threat of disclosing HIV status | Using someone's HIV status as a leverage for blackmail or publicly disclosing it without consent | *The survivor, confiding in his sister about his HIV-positive status, faced discrimination when she disclosed this information to her husband, resulting in their avoidance of personal contact and threat of revealing his status* |
|  |  | Criminalization of MSM | Practices that criminalize consensual same-sex relationships among men | *The coercion and humiliation experienced during a police interrogation, followed by a court ruling under Article 120 of the Criminal Code of Uzbekistan* |
|  |  | Criminalization of HIV transmission | Practices that specifically criminalize the transmission of HIV | *After being discharged from military service due to his HIV status, the survivor was summoned by the police and informed that a criminal case had been initiated against him for allegedly transmitting HIV to other soldiers* |
|  | Freedom from Arbitrary Deprivation of Liberty | Illegal arrest or detention | Detaining individuals without due process, often based on their SOGI | *A transgender woman was illegally detained by police without any reasons provided* |
|  |  | Coercion to provide self-incriminating statements | Forcing individuals to admit to crimes or statements that could incriminate them under duress | *Law enforcement authorities coerced the survivor to provide self-incriminating statements by using psychological and physical pressure tactics, exploiting Article 120 as manipulation to extract confessions, and threatening to create and distribute intimate homosexual videos/photos involving him* |
|  | Freedom from Torture and Cruel, Inhuman or Degrading Treatment or Punishment | Other abuses of authority by security and law enforcement | Various forms of abuse committed by law enforcement officials beyond physical violence | *A group of individuals assaulted a transgender woman, and despite seeking police assistance, she was further abused by law enforcement officers before being taken to the police station* |
|  |  | Force, torture, or cruelty by security and law enforcement | Direct acts of torture or extreme cruelty executed by law enforcement as a means to punish, control, or extract information from individuals | *The student endured prolonged detention, relentless interrogation, physical violence, and threats of imprisonment by security forces during an inquiry about his residency status, spanning from 9:00 a.m. to 1:00 a.m.* |
|  | Work | Employment termination or denial | Being fired from a job or denied employment due to one's SOGI and/or HIV status | *A gay man was denied employment after an interview where his personal relationships were discussed* |
|  |  | Workplace bullying | Experiencing ongoing harassment and intimidation at work related to one's SOGI | *The gay employee, working in a supermarket, faced workplace bullying and harassment from colleagues upon learning about his friendships with transgender people, leading to his dismissal* |
|  |  | Denial to pay wages | Withholding wages from an employee specifically because of their SOGI or HIV status | *The survivor was denied wages after his supervisor discovered his sexual orientation on a dating profile, resulting in additional unwarranted penalties and fines.* |
|  |  | HIV certificate demand during employment | Requiring employees to present an HIV-negative certificate to retain or obtain employment, potentially used discriminatorily | *The survivor seeking employment was asked by the director for HIV and STI certificates based on his appearance* |
|  | Social Security and to Other Social Protection Measures | Denial of benefits or state assistance | Refusing state-provided social benefits or assistance based on an individual's SOGI | *An LGBT activist was denied state social assistance because of his activism* |
|  | Adequate Housing | Eviction or forced eviction | Forcibly removing individuals from their homes due to their SOGI | *A homosexual couple was evicted from their rented apartment following 'complaints from neighbors'* |
|  |  | Denial of hotel and shelter services | Refusing accommodation services to individuals based on their SOGI | *Upon being forcibly relocated to another city, the survivor sought shelter in state-provided facilities, including ordinary schools, where they were explicitly refused accommodation, citing their affiliation with MSM* |
|  | Education | Bullying in educational institutions | Targeted harassment and intimidation of individuals in educational settings due to their SOGI | *In school, a student was systematically teased and pushed because of the way he dressed* |
|  | Highest Attainable Standard of Health | Demeaning conduct in HCF | Disrespectful or humiliating treatment by healthcare providers towards patients based on their SOGI or HIV status | *The survivor, seeking medical assistance from a surgeon, was denied treatment for a pararectal fistula despite a referral, with the surgeon dismissing the need for surgery due to the survivor's HIV-positive status and exhibiting rude behavior upon subsequent visits* |
|  |  | Denial to provide medical services | Refusing healthcare services to individuals because of their SOGI or HIV status | *The survivor sought medical attention for heart pain, only to be refused examination twice and subjected to derogatory remarks about his sexual orientation* |
|  |  | Disclosure of SOGI medical data | Unauthorized sharing of medical data relating to an individual's SOGI | *During a medical consultation, the surgeon, upon learning about the client's HIV testing history and sexual orientation, responded with derogatory remarks and forcibly removed the survivor from the clinic, exposing information about SOGI to other clinic visitors* |
|  |  | Disclosure of HIV medical data | Unauthorized sharing of an individual's HIV status | *A clinic breached privacy by contacting former neighbors of a survivor living with HIV, leading to the dissemination of their HIV status to new tenants, parents, and partners* |
|  |  | Denial of medical services related to transgender transition | Refusing medical care necessary for gender transition | *The transgender woman encountered discrimination when her surgeon abruptly ceased communication and exhibited derogatory behavior upon learning about her transgender identity during a phone conversation* |
|  |  | Denial to prescribe PrEP | Refusing to prescribe HIV prevention medication based on discrimination | *Despite being eligible, the survivor was denied PrEP with discriminatory comments insinuating that PrEP was not intended for gay* |
|  |  | Extortion of payment for medical services, whether free or paid | Demanding unauthorized payments for medical services | *Despite the survivor living with HIV, he was intentionally prescribed costly medications, compelling him to pay for his treatment, despite being entitled to receive it without charge* |
|  |  | Denial of hepatitis or STI treatment | Refusing treatment for hepatitis or sexually transmitted infections based on discriminatory grounds | *The survivor registered in an HIV dispensary, faced derogatory remarks from a substitute doctor who refused to provide a referral for an STI examination* |
|  |  | Denial to prescribe ART | Refusing to provide antiretroviral therapy necessary for treating HIV | *Despite testing positive for HIV at the AIDS Center, the survivor was denied medication due to their lack of residency status in the country* |
|  | Protection from Medical Abuses | Forced anal examinations | Conducting anal examinations without consent to determine sexual behavior | *During her visit to the military enlistment office, a trans*woman endured a degrading anal examination, accompanied by ridicule and mockery from medical staff* |
|  |  | Forced HIV testing | Conducting HIV tests without an individual's consent | *Without consent, a survivor in a hospital underwent HIV testing followed by intrusive questioning about their sexual history and orientation by a physician, resulting in an unwarranted disclosure of their HIV status and discriminatory remarks* |
|  |  | Forced medical treatment | Administering medical treatment without the consent of the individual | *While in a psychiatric clinic, the transgender woman was subjected to conversion therapy attempts by her doctor.* |
|  | Freedom of Opinion and Expression | Hate speech in media and public figures | Derogatory or inciting speech against LGBT individuals by media outlets or public figures | *The blogger used derogatory language in multiple live broadcasts on platforms like YouTube and Facebook, specifically targeting LGBT organization for their advocacy of LGBT rights* |
|  |  | Hate speech and public incitement by individuals | Expressions that incite violence or discrimination against LGBT individuals by private individuals | *While at a sex shop, a heterosexual couple subjected a survivor to derogatory remarks, threats, and hateful language due to his sexual orientation. The couple openly ridiculed him and threatened to expose his affiliation to his community.* |
|  | Freedom of Peaceful Assembly and Association | Obstacles in the work of non-governmental organizations | Interference or restrictions that hinder the operations of NGOs working for LGBT rights | *An LGBT organization leased office space in a business center but faced opposition from other tenants upon revealing its services for the LGBT community and HIV testing, leading to an unjust eviction demand from the landlord.* |
|  |  | Obstacles in conducting meetings | Impediments to organizing or conducting gatherings for LGBT rights advocacy | *Transgender activists faced obstruction from security guards, as they attempted to take photos with an LGBT flag, resulting in their forceful ejection and subsequent police refusal to intervene, citing insufficient evidence* |
|  | Freedom of Movement | Forced departure from city / country | Compelling individuals to leave their residence or country due to their SOGI | *MSM survived a violent altercation with his parents, only to endure ongoing abuse, but his situation worsened when he was coerced into labor abroad in Korea, against his wishes* |
|  |  | Denial to cross borders | Preventing individuals from crossing country borders based on their SOGI | *Despite identifying as a transgender woman, the survivor was refused border crossing due to a mismatch between her appearance and the gender marker in her passport* |
|  | Found a Family | Coercion into marriage | Forcing individuals to enter into heterosexual marriages against their will as a way to conceal their SOGI | *In response to his revealed homosexuality, a survivor faced familial coercion and physical violence, ultimately culminating in his forced marriage to a woman* |
|  | Participate in Public Life | Denial of employment or dismissal from public service | Discriminatory non-hiring or firing from public sector jobs based on SOGI | *After his colleagues and superiors at the city education department discovered private conversations on his work computer, a gay employee was forcibly fired* |
|  |  | Denial of employment in Security and Law Enforcement | Discriminatory practices preventing employment in security and law enforcement based on SOGI | *A police officer was forced to resign due to being gay, as his supervisor believed it brought dishonor to the uniform.* |
|  | Effective Remedies and Redress | Denial to provide legal assistance | Withholding legal support or representation in cases involving LGBT rights violations | *After a transgender woman was assaulted and received death threats, the police failed to investigate or provide any assistance to the survivor* |
